# Leftover opioids following adult surgical procedures: a systematic review and meta-analysis

**DOI:** 10.1186/s13643-020-01393-8

**Published:** 2020-06-11

**Authors:** Lori Schirle, Amanda L. Stone, Matthew C. Morris, Sarah S. Osmundson, Philip D. Walker, Mary S. Dietrich, Stephen Bruehl

**Affiliations:** 1 School of Nursing, Vanderbilt University, 461 21st Avenue South, Nashville, TN 37240 USA; 2 Department of Anesthesiology, Vanderbilt University Medical Center, Nashville, TN USA; 3 Department of Psychiatry and Human Behavior, University of Mississippi Medical Center, Jackson, MS USA; 4 Department of Obstetrics & Gynecology, Vanderbilt University Medical Center, Nashville, TN USA; 5 Eskind Biomedical Library, Vanderbilt University, Nashville, TN USA; 6 Department of Biostatistics, Vanderbilt University Medical Center, Nashville, TN USA

**Keywords:** Postoperative pain, Postoperative care, Opioid usage, Acute pain, Pain medications, Outpatient

## Abstract

**Background:**

US opioid prescribing and use escalated over the last two decades, with parallel increases in opioid misuse, opioid-related deaths, and concerns about diversion. Postoperatively prescribed opioids contribute to these problems. Policy makers have addressed this issue by limiting postoperative opioid prescribing. However, until recently, little data existed to guide prescribers on opioid needs postoperatively. This meta-analysis quantitatively integrated the growing literature regarding extent of opioids leftover after surgery and identified factors associated with leftover opioid proportions.

**Methods:**

We conducted a meta-analysis of observational studies quantifying postoperative opioid consumption in North American adults, and evaluated effect size moderators using robust variance estimation meta-regression. Medline, EMBASE, Cumulative Index of Nursing and Allied Health Literature, and Cochrane Database of Systematic Reviews were searched for relevant articles published January 1, 2000 to November 10, 2018. The Methodological Index for Non-Randomized Studies (MINORS) tool assessed risk of study bias. The proportion effect size quantified the primary outcome: proportion of prescribed postoperative opioids leftover at the time of follow-up. Primary meta-regression analyses tested surgical type, amount of opioids prescribed, and study publication year as possible moderators. Secondary meta-regression models included surgical invasiveness, age, race, gender, postoperative day of data collection, and preoperative opioid use.

**Results:**

We screened 911 citations and included 44 studies (13,068 patients). The mean weighted effect size for proportion of postoperative opioid prescriptions leftover was 61% (95% CI, 56-67%). Meta-regression models revealed type of surgical procedure and level of invasiveness had a statistically significant effect on proportion of opioids leftover. Proportion of opioids leftover was greater for “other soft tissue” surgeries than abdominal/pelvic surgeries, but did not differ significantly between orthopedic and abdominal/pelvic surgeries. Minimally invasive compared to open surgeries resulted in a greater proportion of opioids leftover. Limitations include predominance of studies from academic settings, inconsistent reporting of confounders, and a possible publication bias toward studies reporting smaller leftover opioid proportions.

**Conclusions and implications of key findings:**

A significant proportion of opioids are leftover postoperatively. Surgery type and level of invasiveness affect postoperative opioid consumption. Integration of such factors into prescribing guidelines may help minimize opioid overprescribing while adequately meeting analgesic needs.

## Background

Despite increasing national attention and concentrated policy efforts, the opioid epidemic continues to grow, claiming 130 lives daily and contributing to an unprecedented recent decrease in life expectancy in the USA (US) [1]. Opioid analgesics are commonly prescribed for acute pain following surgical interventions [2], with over one million surgical procedures performed annually in the US [3]. Wide variations in opioid prescribing across providers and in opioid consumption across patients can result in a significant proportion of leftover opioids following surgery [4, 5, 6]. For example, one study reported discharge opioid prescriptions ranging from zero to 100 pills after laparoscopic cholecystectomy, while patients consumed on average less than 10 pills [5]. Given that more than half of individuals who misuse prescription opioids obtain them from a friend or relative’s supply [7], leftover opioids following surgery represent a significant public health issue. Beyond diversion concerns, larger quantities of opioids prescribed following surgery have been associated with increased opioid consumption [6, 8, 9]. In turn, evidence suggests that greater postsurgical opioid consumption may contribute to long-term opioid use [10, 11] and development of opioid use disorders [12].

Recent policy initiatives enacted by states, insurers, and pharmacies have sought to decrease opioid diversion and misuse by placing limits on opioid quantities prescribed after surgical procedures [13]. Although these efforts may decrease the absolute number of opioids leftover by patients, considerable quantities of opioids may nonetheless remain unused in patients who consume few to no opioids after surgery. Conversely, these policies may cause unintended harm to patients requiring larger amounts of opioids for adequate pain control, as poorly managed postoperative pain is a major risk factor for developing chronic postsurgical pain [14].

One reason cited for variation in opioid prescribing practices is the lack of adequate data-driven knowledge about analgesic needs after surgery to guide clinician opioid prescribing [15]. This knowledge gap is particularly important to address given the opioid prescribing policy changes currently being enacted. Over the past several years, a growing number of studies have evaluated surgery-specific opioid consumption patterns, although these data have yet to be integrated quantitatively. A 2017 qualitative systematic review summarized six studies addressing postoperative opioid consumption, and reported that 42-71% of prescribed opioids remain unused, with most stored in unsecure locations [16]. A second qualitative review published several months later identified 11 studies addressing postoperative opioid consumption, and reported similar findings [17]. The aim of the current meta-analysis is to quantitatively integrate for the first time the rapidly growing literature regarding extent of leftover opioids after surgery and identify factors associated with the amount of leftover opioids. A primary meta-regression model evaluated factors that may be linked to extent of leftover opioids following surgery, including surgical type, amount prescribed, measurement method, changes in prescribing patterns over time, and geographic region. Secondary meta-regression models also evaluated the influence of demographic variables, surgical invasiveness, use of opioids at the time of surgery, and timing of postoperative opioid consumption data collection. Based upon previous research, our primary hypothesis was that a substantial percentage of opioids prescribed would be leftover [16, 17], and that orthopedic surgeries would result in fewer leftover opioids due to higher pain intensity related to the greater bone/soft tissue disruption involved [18, 19].

## Methods

**Fig. 1:**
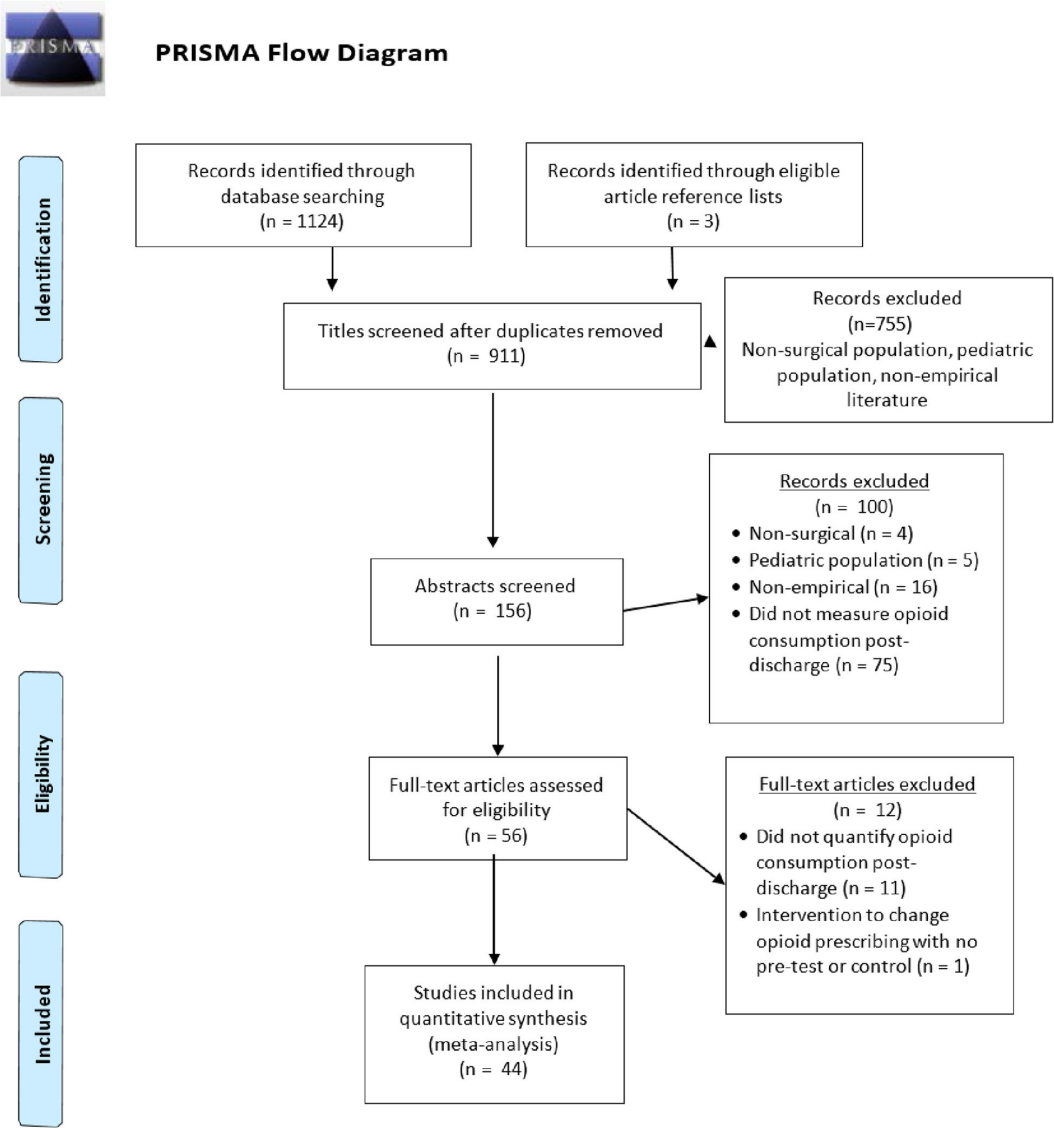
PRISMA flow diagram

This meta-analysis was conducted according to the Preferred Reporting Items for Systematic Review and Meta-Analysis (PRISMA) guidelines (Fig. 1). The PRISMA checklist can be found in supplemental documentation [20]. No prior protocol was published for this project.

### Eligibility criteria

We included studies in adult surgical populations of any design published in North America that reported both the amount of opioids prescribed and consumed for the postsurgical period after patient discharge. We limited our search to North America, as beliefs about opioid prescribing and pain control expectations vary between countries and regions [21], and the US and Canada are the top opioid consuming nations, with similar pharmaceutical industry influences [22]. Exclusion criteria for the current meta-analysis were (a) significant presence of pediatric patients (> 5% of study population), (b) only reported inpatient opioid consumption, (c) did not quantify opioid prescriptions and consumption by morphine milligram equivalents (MME) or number of pills, and (d) use of an intervention that would affect opioid consumption patterns, as interventions would obscure the natural variations in opioid use that were the focus of this review (no-intervention control conditions in studies testing an intervention were included when available).

### Data sources

Medline (via PubMed), EMBASE (OvidSP), Cumulative Index of Nursing and Allied Health Literature (CINAHL) (EBSCOhost), and the Cochrane Database of Systematic Reviews (Wiley) were searched for relevant articles published from January 1, 2000 to November 10, 2018. The final search was performed on December 10, 2018. Online biomedical literature databases were searched by using a combination of keywords and database-specific subject headings determined by a biomedical librarian (P.W.) who has expertise in biomedical literature searches. The search strategy is available in supplemental documentation. Reference lists from eligible studies and prior review articles on the topic were scanned for other eligible studies that may have been missed by search criteria.

### Data extraction and quality assessment

Two authors (A.S. and L.S.) independently extracted relevant study data using a data extraction template. Extracted data included surgery type (abdominal/pelvic, orthopedic, other soft tissue), invasiveness (minimally invasive, open), sample demographic characteristics (sex, race, age), geographic region based upon US Census-designated region s[23] (1, Northeast; 2, Midwest; 3, South; 4, West; 5, Multiple Regions; 6, Canada) of data collection, year of publication, aggregate amount of opioid prescribed (converted to morphine milligram equivalents-MME [24]) and consumed per surgical type, timing of opioid consumption data collection relative to surgery, preoperative opioid use, and type of summary statistic for prescribed and consumed opioids reported (mean or median). These factors were chosen based on a preliminary review of eligible studies and the data available as well as hypothesized factors which could affect opioid consumption patterns. Attempts were made to contact authors of selected studies to supply key missing data (supplementary documentation). Given the observational nature of all studies included in this meta-analysis (Level II and III evidence only), two authors (A.S. and L.S.) independently provided a detailed assessment of the quality of studies using the Methodological Index for Non-Randomized Studies (MINORS) risk of bias tool [25] and demonstrated adequate agreement (87.2%). Disagreements on data extraction or quality ratings were resolved by discussion and consensus or consultation of a third author (M.D.).

### Data analytic plan

Statistical analyses were conducted in the R statistical environment (version 3.6.1). The proportion effect size (*ES*_*P*_) was used to determine the proportion of postsurgical opioids prescribed that was leftover at the time of follow-up. Proportions were computed as the amount of prescribed opioids (mean/median number of pills/MMEs) remaining at post-surgery assessment (numerator) divided by the amount of opioids (mean/median number of pills/MMEs) that were originally prescribed (denominator). Effect size means and variances were estimated from studies reporting medians using established methods [26]. Due to the approximately normal distribution of observed proportions, no transformation of the distribution of *ES*_*P*_ was required [27]. An innovative technique known as robust variance estimation (RVE) meta-regression was used to handle statistically dependent effect sizes (i.e., multiple effect sizes nested within studies) [28]; RVE was implemented in R using the *robumeta* [29] and *clubSandwich* [30] packages. RVE analyses included small sample adjustments for *t* tests. Two-level mixed effects models were specified to allow simultaneous estimation of within-study (level 1) and between-study (level 2) parameters. The intra-class correlation used to calculate variance components in the random effects model (*ρ*) was set at 0.8. The proportion of observed variation across studies that is due to true effects—rather than sampling error—was assessed with the *I*^2^ statistic [31, 32]. High *I*^2^ values (defined as greater than or equal to 75% [33]) suggest that the proportion of opioids leftover likely depends on moderators. The *I*^2^ statistic is a relative measure and does not reflect the absolute amount of heterogeneity [33]. To better capture dispersion, effect sizes are reported with 95% confidence intervals.

RVE meta-regression models tested effect size moderators. All studies reviewed had data available for the primary moderators of interest: surgical type (categorical: abdominal/pelvic, other soft tissue, and orthopedic), amount of opioids prescribed (in MMEs), and study publication year. To address methodological variability and maximize generalizability of results, the meta-regression models statistically controlled for effect size measurement method (mean versus median), publication year, and for the geographic region in which data were obtained. Secondary meta-regression models were conducted for the following potential moderators given their availability in only a subset of studies: surgical invasiveness (open vs. minimally-invasive), age, race, gender, postoperative day of data collection, and preoperative opioid use. All models were evaluated for possible multi-collinearity, and no issues were noted. A type I error rate of 0.05 was used for assessing statistical significance (i.e., *p* < 0.05).

## Results

### Study selection and characteristics

The initial search criteria identified 911 unique citations (Fig. 1) [20]. After an initial screening of titles and abstracts, 156 full-text articles were assessed for eligibility. Of these articles, 44 studies met eligibility criteria and were included in the final analysis (Table 1). Publication dates ranged from 2004-2018 with > 80% published in 2017 or later. Studies represented a broad variety of surgeries ranging from those with minimal tissue disruption (e.g., carpal tunnel repair) to those with major bone and tissue disruption (e.g., spinal fusion). The majority of studies were conducted at single academic medical centers in the Eastern US on predominately Caucasian populations and used a prospective observational cohort design with moderate risk of bias (Table 1). Most studies obtained opioid prescription data through electronic health record review or patient report, and opioid consumption data through patient report via phone, electronic, or in-person survey. A small number of studies used observational methods (e.g., pill count) to reduce self-report bias. Studies varied in their methods for reporting (i.e., number of pills vs. MMEs) and summarizing (i.e., mean vs. median) opioid consumption (Table 1). Of the 44 studies included, 3 were deemed at high risk of bias, 29 were deemed at medium risk of bias, and the remaining 12 studies were deemed at low risk of bias. The primary risks of bias detected were lack of an a priori power calculation to determine sample size, presence of bias for the endpoint (e.g., study conducted by authors who were primary prescribing physicians in the study), and low response rates to follow-up surveys. RVE meta-regression did not reveal a significant overall effect of study bias risk ratings (1, low risk; 2, medium risk; 3, high risk) on proportion of prescribed opioids leftover (*b* = 0.04, *SE* = 0.05, *p* = 0.42, 95% CI, −0.06, 0.15).

**Table 1:** Studies included in meta-analysis

Study	Surgery type	Sample size	Mean (age)	Female (%)	Sample with pre-operative opioid use (%)	Region	Opioid outcome	Method	Postop day	Risk of bias
Alter and Ilyas [48]	Carpel tunnel release	20	62	70	0	Midwest	Pills	Mean	3	L
As-Sanie, et al. [49]	Hysterectomy	102	49	100	12	Midwest	MME	Median	14	L
	Minimally invasive	86								
	Open	16								
Bateman, et al. [8]	Cesarean section	615	33	100		Multiple	Pills	Median	14	L
Bates et al. [50]	Urologic surgery	226				West	Pills	Mean	28	M
Beck et al. [51]	ACL reconstruction	125	33.5	48		Midwest	Pills	Mean	7	M
Cabo et al. [52]	Urologic surgery	77	62.9	35	16	South	MME	Median	21	M
Chapman et al. [53]	Carpal tunnel release	277	64.6	56		Northeast	Pills	Mean	--	H
Cunningham et al. [54]	Hip arthroscopy	73	36.5	75	22	South	Pills and MME	Mean	42	L
Fujii et al. [4]	Various surgeries	333	58			Northeast	MME	Median	7	M
	Partial mastectomy;	21	62.9	96	0					
	Umbilical hernia repair (open)	16	49	42	5					
	Appendectomy (lap)	17	41.6	56	0					
	Cholecystectomy (lap)	40	51.3	70	2.5					
	Inguinal hernia repair (open)	25	59.5	0	0					
	Carpal tunnel release	28	60.8	63	0					
	Knee arthroscopy	30	42	47	3					
	Shoulder arthroscopy	23	57.4	36	12					
	Total hip arthroplasty	45	61.3	48	20					
	Total knee arthroplasty	31	65	56	6					
	Vasectomy	11	38.8	0	0					
	Endoscopy	29	58.5	48	19					
	Prostatectomy (lap)	17	62.8	0	0					
Griffith et al. [55]	Hysterectomy	53				Northeast	Pills	Mean	300	M
	Minimally invasive	39	60.6	100						
	Open	14	32	100						
Gupta et al. [56]	Foot and ankle surgery	84	47	66	0	Northeast	Pills	Mean	14	M
Harris et al. [57]	Dermatologic surgery	212	68	28		West	Pills	Mean	4	M
Hart et al. [58]	Breast surgery	95	60.4	100		South	Pills	Mean	30	H
Hartford et al. [59]	Abdominal surgery	171	50	47		Canada	Pills and MME	Mean	21	L
	Cholecystectomy (lap)	84								
	Open hernia repair	87			0					
Hill et al. [5]	Various surgeries	127			0	Northeast	Pills	Median	--	M
	Partial mastectomy	20			0					
	Partial mast/node biopsy	21			0					
	Cholecystectomy (lap)	48			0					
	Inguinal hernia repair (lap)	20			0					
	Inguinal hernia repair (open)	18			0					
Hill et al. [60]	Various surgeries	234		63	0	Northeast	Pills	Mean	--	M
	Bariatric	83			0					
	Foregut	33			0					
	Hepatectomy	12			0					
	Pancreatectomy	10			0					
	Colectomy	69			0					
	Ventral hernia	27			0					
Hota et al. [61]	Gynecologic and pelvic reconstructive surgery	51	53	100		Northeast	Pills	Median	14	M
	Major	2								
	Minor	49								
Howard, Fry et al. [6]	Various surgeries	2392				Midwest	Pills and MME	Median	30-120	M
	Cholecystectomy (lap)	603	51.2	71						
	Appendectomy (lap)	224	44.6	53						
	Inguinal and femoral hernia repair (open or lap)	659	58	19						
	Open incisional hernia repair	159	60.1	57						
	Colectomy (lap)	112	63.5	56						
	Open colectomy	102	64.7	55						
	Ileostomy and colostomy takedown	59	57.9	42						
	Small-bowel resection and/or enterolysis	33	68	58						
	Thyroidectomy	40	56	83						
	Vaginal hysterectomy	113	51.7	100						
	Hysterectomy (lap)	203	49.7	100						
	Abdominal hysterectomy	85	51.5	100						
Howard, Waljee et al. [62]	Cholecystectomy (lap)	100	46	78		Midwest	Pills and MME	Median	< 365	H
Ilyas et al. [63]	Carpal tunnel release	37				Northeast	Pills	Mean	5	L
	Minimally invasive	19	59	42	0					
	Open	18	61	67	0					
Kim et al. [15•]	Upper extremity surgery	1392	56	55		Northeast	Pills	Mean	Postop visit	L
	Bone	499								
	Soft tissue	893								
Kumar et al. [64]	Shoulder surgery	62	48	26	0	Northeast	Pills	Mean	90	L
Maughan et al. [65]	Dental surgery	72	28	61	0	Northeast	Pills and MME	Median	21	M
Merrill et al. [66]	Foot and ankle surgery	132	53.2	62	0	South	Pills	Mean	10	M
	Bone	91								
	Soft tissue	41								
Miller et al. [67]	Carpal tunnel release	159	59.4	56	0	Northeast	Pills	Mean	--	M
Osmundson et al. [68]	Cesarean section	179	31	100		South	Pills and MME	Median	14	M
Osmundson et al. [9]	Cesarean section	85	30	100		South	Pills and MME	Median	14	L
Patel HD et al. [69]	Radical prostatectomy	205				South	MME	Mean	30	L
	Minimally invasive	149	61.5	0						
	Open	56	61.2	0						
Patel S et al. [70•]	Rhinoplasty	62				Multiple	Pills	Mean	5	M
Peters et al. [71]	Carpal tunnel release	49	57	66	0	Canada	Pills	Mean	84	M
Riley et al. [72]	Rhinologic surgery	42	48.6	33	2.4	Northeast	MME	Median	14	M
Rodgers et al. [73]	Upper extremity surgery	249	54	67	0	Midwest	Pills	Mean	14	M
	Bone	58								
	Soft tissue	191								
Sabatino et al. [74]	Orthopedic procedures	557				Northeast	Pills	Mean	14	M
	Total hip arthroplasty	198	65	52	19					
	Total knee arthroplasty	146	65.7	56	17					
	Carpal tunnel release	91	59.6	58	15					
	Rotator cuff repair	72	60.6	32	17					
	Lumbar decompression	50	65.6	48	20					
Saini et al. [75•]	Foot and ankle surgery	988	49	62	0	Northeast	Pills	Median	14	M
	Bone	754								
	Soft tissue	234								
Schmidt et al. [76]	Cesarean section	141	32	100		Midwest	Pills	Median	14	M
Solouki et al. [77]	Urogynecologic surgery	143	59.5	100	12.6	Northeast	Pills	Mean	30	M
Stepan, et al. [78]	Hand surgery	54	54.9	57	0	Midwest	MME	Mean	7	L
Swarup et al. [79]	Anorectal surgery	23		48	0	Northeast	Pills	Median	14	M
Swenson et al. [80]	Urogynecologic surgery	50	63	100	20	Midwest	Pills	Median	14	M
Tan et al. [81]	Abdominal procedures	176	60.4	50	0	Midwest	MME	Median	26	M
	Minimally invasive	123								
	Open	53								
Tharakan et al. [82]	Thyroidectomy and parathyroidectomy	89	55	82		Northeast	Pills	Median	42-730	M
Thiels, et al. [83]	Various surgeries	2486				Multiple	MME	Median	28	M
	Carotid endarterectomy	73	73	37						
	Parathyroidectomy	108	67.5	83						
	Arteriovenous fistula creation	63	68	47						
	MIS partial colectomy with anastomosis	70	62	68						
	Carpel tunnel release	128	65	100						
	Breast lumpectomy	111	65	58						
	MIS cholecystectomy	138	64	16						
	MIS inguinal hernia repair	107	65	100						
	Ovarian cancer cytoreduction	58	62	6						
	Open inguinal hernia repair	109	70	97						
	Simple mastectomy	76	67.5	100						
	MIS hysterectomy;	139	56	36						
	MIS low anterior resection;	25	59	0						
	MIS prostatectomy	105	62	39						
	MIS nephrectomy	100	65	47						
	Knee arthroscopic meniscectomy	112	49	45						
	Open pancreaticoduodenectomy	40	65.5							
	MIS lung wedge resection	110	64.5	49						
	Tonsillectomy	60	31	70						
	Rotator cuff surgery	129	61	36						
	Lumbar Laminotomy/laminectomy	91	60	32						
	Open lung lobectomy	43	66	49						
	Lumbar fusion	75	59	41						
	Total hip arthroplasty	202	67	48						
	Total knee arthroplasty	214	69	53						
Weiland et al. [84•]	Third molar extraction	48	19.6	67		Northeast	Pills	Median	7	M
Wojahn et al. [85•]	Knee arthroscopy	221	46.2	48	2.7	Midwest	Pills	Median	42	L

Risk of bias scores based on MINORS tool [19]. Studies were rated on 8 categories: (1) clearly stated aim, (2) inclusion of consecutive patients, (3) prospective collection of data, (4) endpoints appropriate to aim or study, (5) unbiased assessment of study endpoint, (6) follow-up period appropriate to aim of study, (7) loss to follow-up rate (adapted to < 20% based on typical study endpoints), and (8) prospective calculation of study size. We added a 9th category, assessment methodology to further adapt the instrument for use for this project. Each category received a 0-2 score. On this scale, 0—not reported, 1—reported, but inadequate, and 2—reported and adequate. Thus, each study received a score between 0-18. Risk of bias categories were assigned based on tertiles: high (H), 0-5; moderate (M), 6-12; low (L), 13-18

### Primary analyses

Across 115 effect sizes drawn from 44 studies (*n* = 13,068 patients), the mean weighted effect size for the proportion of prescribed postsurgical opioids leftover at follow-up was 61% (*t* = 23.1, *df* = 42.5, 95% CI, 56-67%). The summary statistics reported in the included studies were weighted by sample size and pooled to estimate that 2,909,744 prescribed MMEs were represented in those studies (i.e., equivalent of 581,949 5 mg hydrocodone tablets). Thus, results across the included studies indicate that a total equivalent of 354,989 5 mg hydrocodone tablets were leftover, or 27 hydrocodone tablets prescribed, but not used per person. The *I*^*2*^ value of 94.9% reveals variation of true effects (as opposed to sampling error) and indicates that the mean weighted effect size for the proportion of prescribed postsurgical opioids leftover may not be the most appropriate estimate for all studies. Instead, this *I*^*2*^ value suggests that the proportion of opioids leftover likely depends on potential moderators, which supports the subsequent use of RVE meta-regression.

**Fig. 2:**
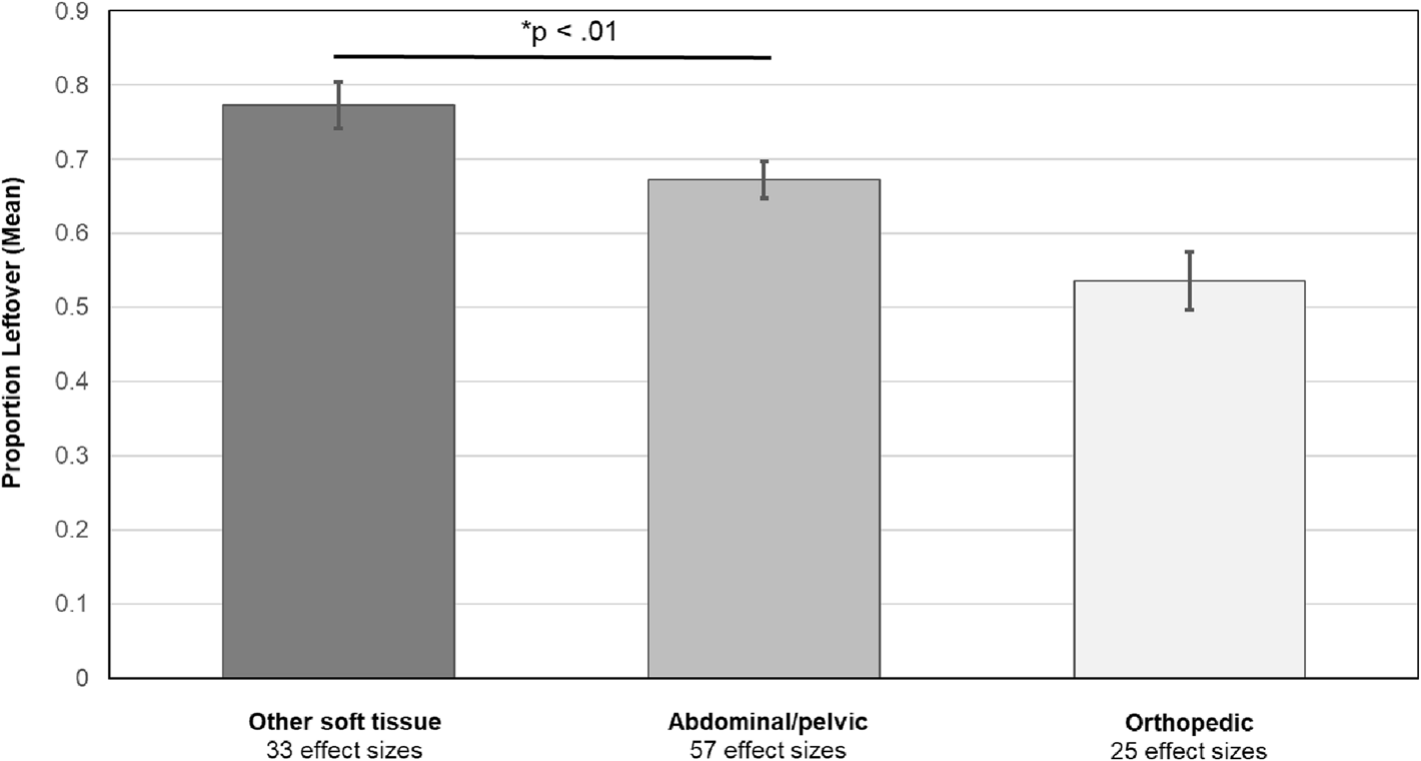
Mean proportions of postsurgical opioid prescriptions leftover (+/−standard error of the mean) by surgical type

Primary RVE meta-regression models revealed a significant overall moderating effect of surgical type on the proportion of opioid prescriptions leftover (*b* = 0.09, *SE* = 0.03, *p* < 0.01, 95% CI, 0.03, 0.15) (Table 2). This model accounted for 34% of the between-study variance. As shown in Fig. 2, specific surgical type contrasts indicated that most of that overall effect was explained by significantly greater leftover opioids for “other soft tissue” surgeries (i.e., chest/breast, head/dental, other soft tissue) than for abdominal/pelvic surgeries (*b* = 0.18, *SE* = 0.05, *p* < 0.01; 95% CI, 0.07, 0.29); proportions did not differ significantly between orthopedic and abdominal/pelvic surgeries (*b* = 0.07, *SE* = 0.07, *p* = 0.36; 95% CI, −0.08, 0.22), nor between “other soft” and orthopedic (*b* = 0.11, *SE* = 0.07, *p =* 0.11; 95% CI, −0.03, 0.25). Orthopedic studies reported significantly more variability in postoperative opioids consumed (coefficient of variation [CV] = 0.37, 95% CI, 0.26, 0.48) versus abdominal/pelvic (CV = 0.28, 95% CI, 0.23, 0.33) or other soft tissue (CV = 0.23, 95% CI, 0.17, 0.29) (both *p* values < 0.05).

**Table 2:** Summary of primary robust variance estimation meta-regression model predicting proportions leftover of postsurgical opioid prescriptions

	*b*	*SE*	*t* value	*df*	95% CI	*p*
Measurement method	0.082	0.053	1.54	24.33	−0.028, +0.192	0.137
Region	−0.006	0.014	0.47	10.50	−0.037, +0.024	0.648
Publication year	0.016	0.007	2.47	2.51	−0.007, +0.040	0.106
Opioids prescribed (MME)	−0.0003	0.0002	1.65	6.40	−0.0009, +0.0002	0.147
Surgical type	0.089	0.027	3.26	23.18	+0.033, +0.146	0.003

Results were based on 115 effect sizes drawn from 44 studies. The intraclass correlation (*ρ*) was set at 0.8

Measurement method (mean, 0; median, 1); region (1, Northeast; 2, Midwest; 3, South; 4, West; 5, Multiple regions; 6, Canada); publication year (centered such that 0, first published study in 2004); surgical type (1, “abdominal/pelvic;” 2, “orthopedic” [including joint/spine, other boney]; 3, “other soft tissue” [including chest/breast, head (dental), other soft tissue])

*b* unstandardized regression coefficient; *SE* standard error; *CI* confidence interval

**Fig. 3:**
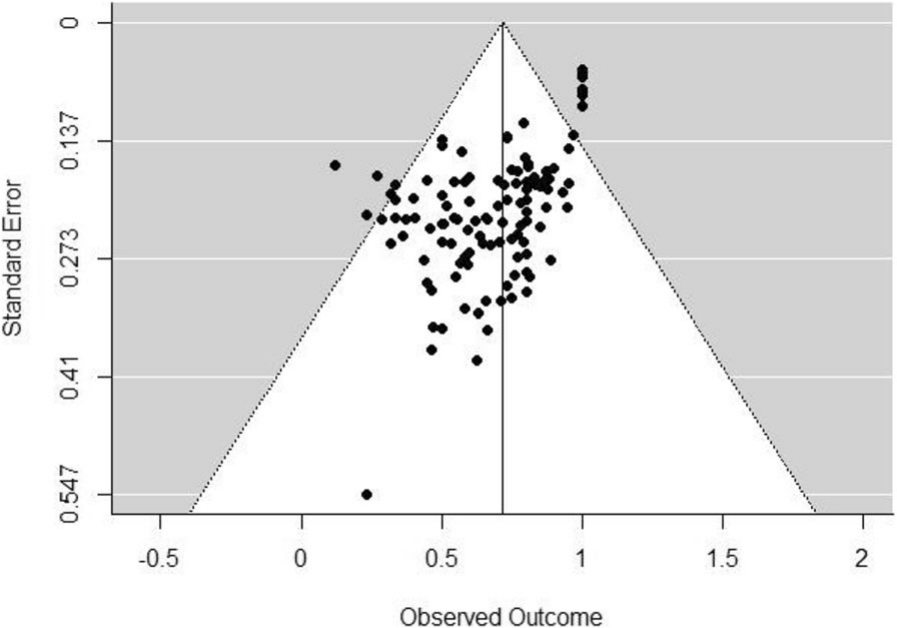
Funnel plot for studies reporting proportions of postsurgical opioid prescriptions leftover

Primary RVE meta-regression models did not reveal significant overall effects of measurement method (*b* = 0.08, *SE* = 0.05, *p* = 0.14, 95% CI, −0.03, 0.19), geographic region (*b* = −0.01, *SE* = 0.01, *p* = 0.65, 95% CI, −0.04, 0.02), publication year (*b* = 0.02, *SE* = 0.01, *p* = 0.11, 95% CI, −0.01, 0.04), or amount of opioids prescribed (*b* = −0.0003, *SE* = 0.0002, *p* = 0.15, 95% CI, −0.0009, 0.0002). A funnel plot displaying the association between effect size estimates and their standard errors is presented in Fig. 3. Egger’s test was significant (*z* = −10.23, *p* < 0.001), indicating funnel plot asymmetry. Visual inspection of this funnel plot revealed a potential publication bias toward studies reporting smaller proportions of opioid prescriptions leftover. Examining separate funnel plots for studies reporting means (*n* = 76) versus medians (*n* = 39, see Supplemental Figure 1) indicated an absence of asymmetry for the means-only plot (*z* = −0.47, *p* = 0.63) but continuing asymmetry for the medians-only plot (z = −7.96, *p* < 0.001). The latter result revealed a trend for larger medians-only studies to report greater proportions of opioids leftover.

### Secondary analyses

**Fig. 4:**
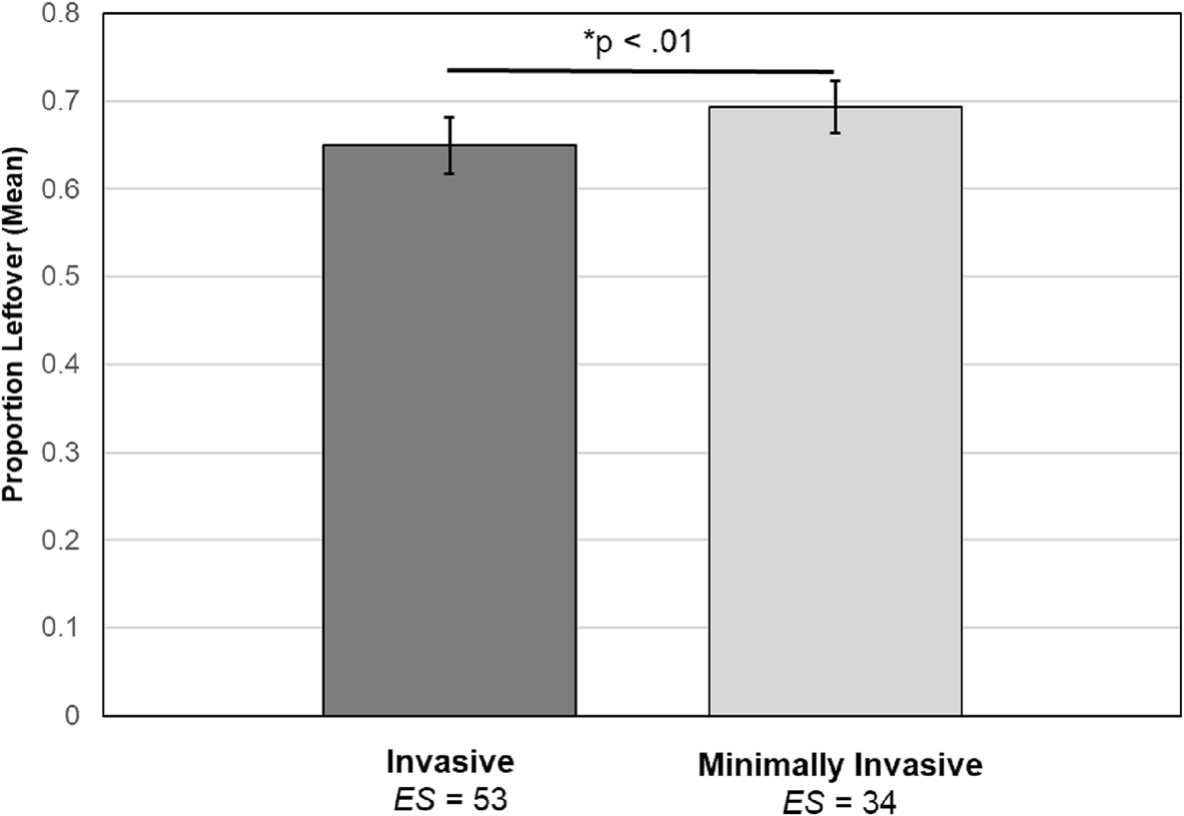
Mean proportions of postsurgical opioid prescriptions leftover (+/−standard error of the mean) for studies reporting invasive versus minimally invasive surgical procedures

Effects of other moderators on the proportion of prescribed opioids leftover following surgery were assessed for the subset of studies that included each. These models all controlled for measurement method, region, publication year, amount of opioids prescribed, and surgery type (i.e., all variables included in the primary analyses). Findings indicated that more invasive open surgical procedures were associated with a significantly lower proportion of prescribed opioids leftover following surgery relative to minimally invasive procedures (*k* = 36 studies, number of effect sizes = 87; *b* = −0.16, *SE* = 0.05, *p* < 0.01, 95% CI, −0.25, −0.06; Fig. 4). None of the following associations were significant: percentage of the sample that was female (*k* = 42 studies, number of effect sizes = 109, *b* = −0.18, *SE* = 0.10, *p* = 0.10, 95% CI, −0.39, 0.03); mean age of sample (*k* = 40 studies, number of effect sizes = 102, *b* = 0.003, *SE* = 0.002, *p* = 0.10, 95% CI, −0.001, 0.008); percentage of the sample that was Caucasian (*k* = 19 studies, number of effect sizes = 51, *b* = 0.21, *SE* = 0.17, *p* = 0.26, 95% CI, −0.21, 0.63); postoperative day of data collection (number of days after surgery opioid consumption data was collected) (*k* = 37 studies, number of effect sizes = 97, *b* = 0.0002, *SE* = 0.0004, *p* = 0.59, 95% CI, −0.002, 0.002); or percentage of the sample using opioids preoperatively (*k* = 26 studies, number of effect sizes = 57, *b* = −0.28, *SE* = 0.53, *p* = 0.61, 95% CI, −1.46, 0.91).

## Discussion

This meta-analysis synthesized data from 44 studies to quantify the extent of leftover opioids following surgery and evaluate factors potentially associated with the proportion of opioids leftover. Overall, we found that 61% of opioids prescribed following surgery were leftover, which amounted to approximately 27 5 mg hydrocodone tablets per person. Assuming a maximum dose of six hydrocodone tablets per day, the average individual was left with enough medication to treat pain for 4.5 additional days. Findings revealed two key moderators associated with proportion of opioids leftover that can guide providers caring for patients after surgery: type of surgery and the degree of invasiveness.

Studies involving surgeries on non-visceral organs (i.e., mastectomy, thyroidectomy) reported significantly more leftover opioids than abdominal/pelvic surgeries, and as expected, minimally invasive techniques were associated with a greater proportion of opioids leftover. Regulatory changes designed to decrease opioid prescribing in these procedures align in part with opioid consumption data from this analysis. For example, Tennessee law limits opioid prescriptions to less than 20 days, depending upon surgery invasiveness [34]. However, patients undergoing abdominal/pelvic surgeries demonstrated fewer leftover opioids relative to other soft tissue procedures (suggesting greater opioid requirements for pain control in abdominal pelvic surgeries). This lack of uniformity across specific soft tissue surgery subtypes is not adequately addressed in the Tennessee prescribing law, potentially contributing to variability in adequacy of pain management. Patients undergoing orthopedic surgeries demonstrated the largest variability in opioid consumption relative to abdominal/pelvic or other soft tissue, potentially leaving these patients more vulnerable to blanket opioid restriction policies. These data highlight the potential harm that could occur with policies that uniformly limit opioid prescribing, and the need to engage broad expertise across specialties in developing opioid prescribing guidelines that are supported by specialty-specific data on opioid consumption. Individual variability noted in opioid use particularly among patients undergoing orthopedic surgeries also highlights the potential value of applying a precision medicine approach to opioid prescribing [35], although data to support this approach are still evolving.

The present study adopted a sophisticated RVE mega-regression method that can handle complex data structures with dependent effect sizes and that applies an adjustment for small sample bias [32]. Nevertheless, these findings should be interpreted with caution in light of potential publication bias (favoring studies reporting smaller proportions of opioids leftover) that was identified based on funnel plot asymmetry. Follow-up analyses indicated that this asymmetry was driven by studies reporting medians which also tended to be the studies with larger sample sizes. Median values would not be influenced by highly skewed distributions. Means being used particularly with small negatively skewed samples would dramatically under-estimate proportions of opioids leftover. Although RVE meta-regression analyses revealed there were no statistically significant differences between studies reporting means compared to medians, the lack of statistically significant differences cannot be interpreted as similarity or equivalence. Standardizing the reporting of opioid consumption will aid further meta-analytic efforts. Based on our review of the data, we recommend reporting medians, interquartile ranges, and absolute ranges for opioids prescribed and consumed due to the skewed nature of the data. Another limitation of our study is the low number of studies conducted in the early years of this analysis (2004-2008), which may limit characterization of opioid prescription and consumption during this period. In addition, studies differed with regard to the inclusion of preoperative opioid users, and inclusion of potential confounders such as chronic pain or mental health conditions known to influence pain. Most of the included studies examined opioid prescribing practices in academic medical settings, which have been found to prescribe more opioids than non-teaching facilities [36]. The extent to which these findings would generalize to non-academic settings is unclear. Finally, this systematic review did not publish an a priori protocol, which might have addressed any concerns about potential bias in its conclusions. The submission of a public protocol allows for peer review of research methods early in the review process, mitigating the potential effects of author biases, and provides readers a tracking mechanism for changes in the review process [37].

Results of this meta-analysis suggest individual variability in the extent of opioids used and consequently leftover postoperatively. Our review indicates that additional research is needed to identify the sources of this variability at a more granular level and in a manner that might be pragmatically useful in a precision medicine context. Studies of this issue to date consistently report only a small number of factors, primarily procedure-related and demographic, that might drive the extent of opioids left unused postoperatively. Although potentially more challenging pragmatically, it would be valuable to obtain preoperative measures of constructs other literature suggests may influence opioid use. Attitudes towards opioid use are one factor that may influence a patient’s actual use of opioids [38]. Negative affect (e.g., depression, anxiety) has also been shown to be predictive of responsiveness to opioids and extent of postoperative opioid use [39–41]. We further note the importance of considering patient-reported pain intensity as a context for interpreting opioid use outcomes. Unfortunately, pain intensity was not reported in some studies, and was inconsistently reported across the other studies (i.e., at differing time intervals, using different rating scales, and addressing differing characteristics—worst, average, current), and therefore could not be examined systematically in our analysis. Recognizing that timing of clinically meaningful measurement of primary endpoints may vary by surgical procedure [42], future work would benefit from consistent inclusion of validated measures of postoperative pain intensity, ideally obtained concurrently with opioid use data. For example, telephone follow-up that assesses both opioid use and pain ratings over the same period of time would enhance interpretation of opioid data. Obtaining these data at a standard time point across studies, such as one-week post-discharge, would also enhance ability to compare opioid data across studies. Clinical practice guidelines developed by an interdisciplinary expert panel recommend that clinicians use a validated pain assessment tool to track responses to postoperative pain treatment [43]. Given likely patient heterogeneity (in terms of cognitive status, education, etc.) in studies of postoperative opioid use, a simple validated pain measure might be optimal. For simplicity and standardization, we recommend a 0-10 numeric rating scale (NRS) anchored with “no pain” and “worst possible pain” for assessing average pain at rest in the past week (consistent with the suggested follow-up period above). NRS intensity ratings are a preferred outcome in pain trials [44], and retrospective ratings of average pain appear to correspond well with diary-based ratings of momentary pain over the same time period [45]. Measures of pain at rest appear largely to parallel alternative measures of pain relief or pain evoked by activity relevant to the specific surgical procedure [46]. Consistent availability of pain intensity data as described above would facilitate meta-analyses that could evaluate whether the degree of leftover opioids is being driven by differences in actual pain experience, or, alternatively, non-pain factors. For example, evidence in the chronic pain context suggests that opioids may be used not only for pain control, but to reduce negative mood [47]. More comprehensive phenotyping of patients in studies of postoperative opioid consumption going forward could significantly enhance the scientific value of future studies on this topic.

## Conclusions

In summary, results of this meta-analysis of 44 studies reveal that 61% of opioids prescribed following surgery remain unused, providing a large quantity of opioids potentially available for diversion. There is, however, variability in the amount of opioids leftover, with non-abdominal soft tissue surgeries having the highest proportion of opioids leftover compared to abdominal/pelvic and orthopedic procedures. Less invasive laparoscopic procedures also are associated with a higher proportion of opioids leftover compared to open surgical procedures. The current findings for the first time document and quantify these differences across a wide-range of studies, and underscore the potential problems with regulatory efforts to broadly limit postsurgical opioid prescribing without adequately considering surgical characteristics. Better data to guide such regulatory changes and data-driven physician education regarding optimal procedure-specific opioid prescribing are both needed to achieve the goal of minimizing leftover opioids while continuing to provide adequate pain management in the postoperative period.

## Supplementary information

**Additional file 1.** Supplemental documentation.

**Additional file 2.** Effect sizes for mean proportions of opioids leftover.

## Data Availability

The datasets during and/or analyzed during the current study available from the corresponding author on reasonable request.

## Abbreviations

*b*: Unstandardized regression coefficient
CI: Confidence interval
CINAHL: Cumulative Index of Nursing and Allied Health Literature
CV: Coefficient of variation
MINORS: Methodological Index for Non-Randomized Studies
MME: Morphine milligram equivalents
PRISMA: Preferred Reporting Items for Systematic Review and Meta-Analysis
RVE: Robust variance estimation
SE: Standard error
US: United States
