# Supplementary material for: Leftover opioids following adult surgical procedures: a systematic review and meta-analysis
Source: Syst Rev. 2020 Jun 11;9:139. doi: 10.1186/s13643-020-01393-8 (PMC7291535; doi:10.1186/s13643-020-01393-8)
Supplement: Supplementary file 1 — Additional file 1. Supplemental documentation. [file 13643_2020_1393_MOESM1_ESM.docx]

# Search Strategies

# Medline (PubMed):

# ("Postoperative Period"[Mesh] OR postoperative[tiab] OR post-operative[tiab] OR post-surgical[tiab] OR "Postoperative Care"[Mesh] OR "Pain, Postoperative"[Mesh] OR postoperative care[tiab] OR postoperative pain[tiab] OR postsurgical[tiab] OR "Surgical Procedures, Operative"[Mesh] OR surgery[tiab] OR surgical procedures[tiab] OR surgical procedure[tiab]) AND ("Analgesics, Opioid"[Mesh] OR opioid[tiab] OR opioids[tiab]) AND ("Drug Prescriptions"[Mesh] OR prescription[tiab] OR prescriptions[tiab] OR prescribe[tiab] OR prescribed[tiab] OR prescribing[tiab]) AND "humans"[MeSH Terms] AND English[lang] AND ("2000/01/01"[PDAT] : "2018/11/10"[PDAT])

# EMBASE (OvidSP):

#
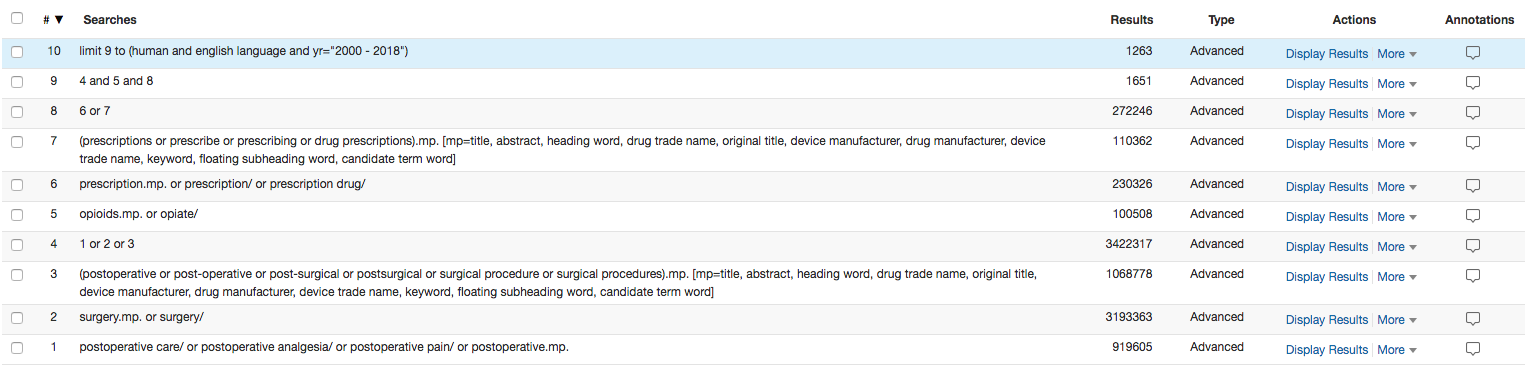


# CINAHL (EBSCOhost):

# (MH "Postoperative Period" OR MH "Postoperative Pain" OR MH "Postoperative Care" OR "postoperative" OR MH "Surgery, Operative" AND "surgery" OR postoperative OR post-operative OR postsurgical OR post-surgical OR postoperative care OR postoperative pain OR surgery OR surgical procedure) AND (MH "Analgesics, Opioid" OR "opioid" OR opioids) AND (MH "Prescriptions, Drug" OR "prescriptions" OR MH "Drugs, Prescription" OR prescription OR prescriptions OR prescribe OR prescribing)

# Limiters - Published Date: 20000101-20181131; English Language; Human

# Cochrane Database of Systematic Reviews (Wiley):

#
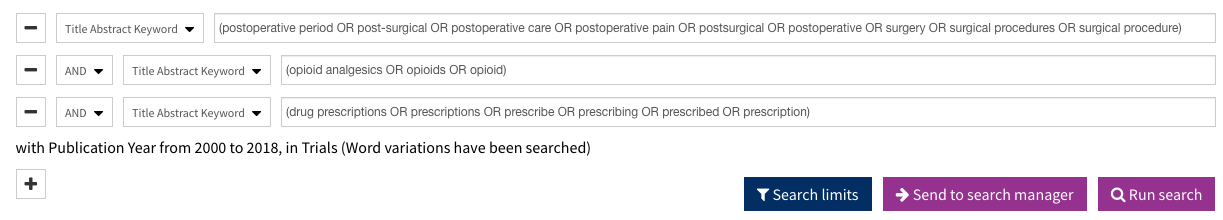


# Unpublished Literature

All conference abstracts and unpublished literature were considered for inclusion if there was evidence postoperative home opioid consumption data was collected at the MME or pill level. One conference abstracts (57) met inclusion criteria during the search period, and were accepted into published manuscript form during the analysis period.

# Additional data

Sixteen authors were contacted to supply missing data. Eleven authors responded with follow up information involving procedure specific demographics or more detailed opioid prescribing or consumption data.
