# Supplementary material for: Leftover opioids following adult surgical procedures: a systematic review and meta-analysis
Source: Syst Rev. 2020 Jun 11;9:139. doi: 10.1186/s13643-020-01393-8 (PMC7291535; doi:10.1186/s13643-020-01393-8)
Supplement: Supplementary file 2 — Additional file 2. Effect sizes for mean proportions of opioids leftover. [file 13643_2020_1393_MOESM2_ESM.docx]

Supplemental Figure 1.


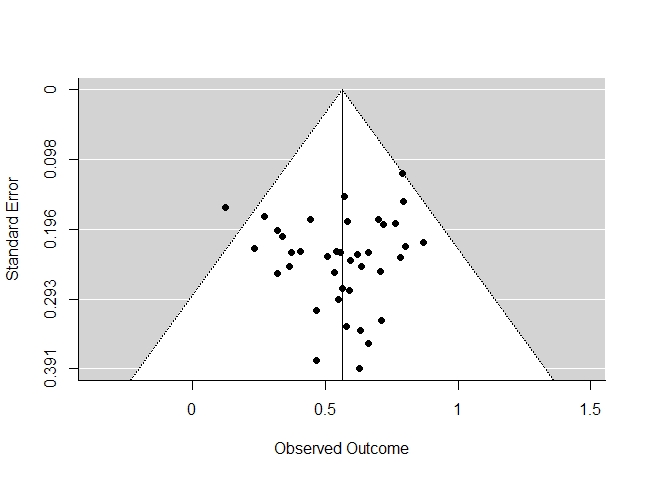


Effect Sizes for Mean Proportions of Opioids Leftover


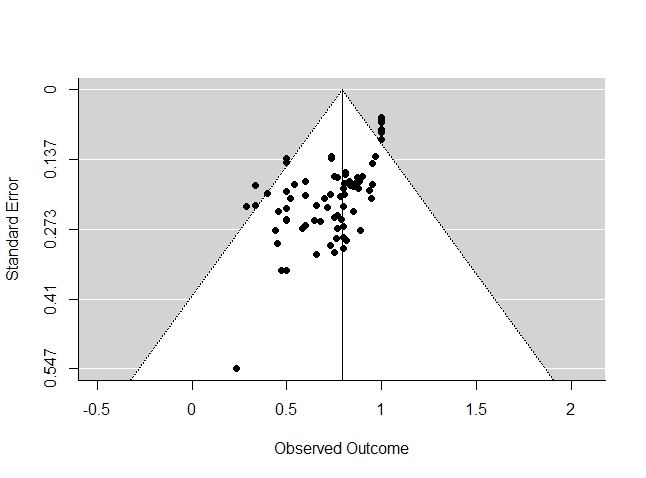


Effect Sizes for Median Proportions of Opioids Leftover
